# Envisioning population‐scale immune multi‐omics atlas projects

**DOI:** 10.1002/ctm2.70693

**Published:** 2026-05-11

**Authors:** Yuhui Zheng, Jianhua Yin, Chuanyu Liu

**Affiliations:** ^1^ State Key Laboratory of Genome and Multi‐omics Technologies BGI Research Shenzhen China; ^2^ College of Life Sciences University of Chinese Academy of Sciences Beijing China; ^3^ Shenzhen Proof‐of‐Concept Center of Digital Cytopathology BGI Research Shenzhen China; ^4^ Shanxi Medical University‐BGI Collaborative Center for Future Medicine Shanxi Medical University Taiyuan China

## 1 THE EVOLUTION OF POPULATION‐SCALE BIOLOGY: BEYOND GENOMIC CATALOGUES

**FIGURE 1:**
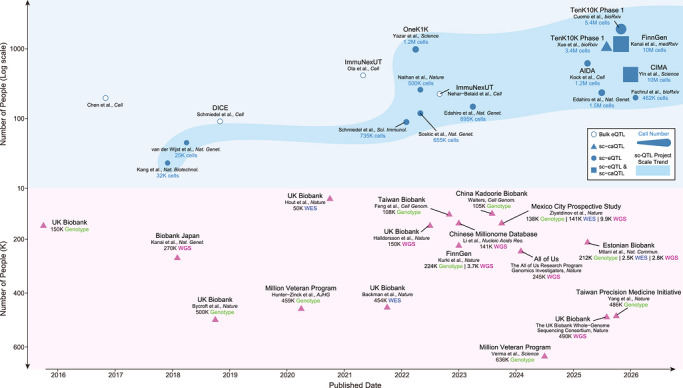
Representative large‐scale population genomics projects and immune cell profiling studies with QTL analyses over the past decade. (Top panel) Immune cell omics studies with QTL analyses. Marker shapes indicate omics modalities, and marker size reflects the number of single cells (annotated). Scaling trends over time are shown. (Bottom panel) Large‐scale population genomics projects. The number of sequenced individuals is indicated for each project, stratified by sequencing technology.

The completion of the Human Genome Project (HGP) in 2003 provided the first reference framework for human genetics and initiated a transition toward population‐scale genomic research.^1^ Over the past decade, this transition has driven the emergence of numerous large‐scale population cohort genomic projects. Among these, the UK Biobank (UKB) stands out not only for its scale but as a benchmark model for such initiatives, integrating large‐scale genotyping with deep phenotyping, longitudinal follow‐up, and open data access.^2^ Notably, UKB also exemplifies a broader technological trajectory observed across the field: an iterative progression from array‐based genotyping,^2^ to whole‐exome sequencing (WES),^3^ and ultimately to whole‐genome sequencing (WGS),^4^ with each successive phase capturing increasing genomic resolution across a growing number of participants (Figure 1 Bottom panel). This framework has profoundly shaped the design of other large‐scale population cohort genomic projects worldwide,^5^ each adapting this paradigm to distinct populations and healthcare systems (Figure 1 Bottom panel).

Together, these initiatives have substantially advanced our ability to associate genetic variation with disease risk across populations, establishing a robust foundation for precision medicine. However, the emphasis on genomic associations has revealed a fundamental limitation: most genetic variants, particularly in non‐coding regions, exert their effects through context‐dependent regulatory mechanisms that are highly cell type‐specific and shaped by chromatin and cellular states.^6^, ^7^ This challenge is amplified in the immune system due to its extreme cellular heterogeneity and dynamic functional responses.^8^ Together, these limitations highlight the need for next‐generation reference frameworks that integrate immune‐specific, multi‐omic, and single‐cell–resolved data beyond population genomics.

## 2 POPULATION‐SCALE IMMUNE MULTI‐OMICS: CONCEPT AND FEASIBILITY

Building on these limitations, the field is witnessing a growing trend toward population‐scale, immune‐focused reference frameworks, giving rise to a new class of initiatives collectively termed Population‐scale Immune Multi‐omics Atlas Projects (PIMAs) (Figure 1 Top panel). Analogous to large‐scale population cohort genomic projects that transformed population genetics, PIMAs seek to systematically characterise immune cell diversity and regulatory states by integrating molecular information across genomic, epigenomic, transcriptomic, proteomic, and metabolic layers at cellular resolution.^7^, ^9^, ^10^ By focusing on coordinated, population‐wide immune profiling rather than isolated datasets, PIMAs provide a foundation for mechanistic interpretation of genetic risk in immune‐mediated diseases.

Recent advances in scalable single‐cell technologies, high‐throughput proteomics, and computational frameworks for multi‐omics integration have enabled the generation of high‐resolution cellular atlases at unprecedented scale, making such population‐scale immune profiling increasingly feasible.^11^, ^12^ Focusing on peripheral blood mononuclear cells (PBMCs) provides a practical and minimally invasive window into systemic immune function, as the diverse repertoire of circulating immune cell subsets they encompass enables near non‐invasive, real‐time characterisation of the organism‐level immune states. These cellular profiles not only capture immune perturbations associated with disease states but also reflect variations driven by genetic background, ancestry, environmental exposures, lifestyle and ageing, thereby serving as scalable and informative readouts for monitoring immune system dynamics across individuals and populations.^13^ Importantly, the cell type‐specific resolution afforded by single‐cell approaches addresses a key limitation of bulk analyses, as immune dysfunction often arises from perturbations in defined cellular subsets that are masked when signals are averaged across heterogeneous cell populations.

Crucially, the comprehensive data generated by such initiatives have the potential to advance the development of virtual cells—data‐driven computational models that integrate multi‐layered molecular information to represent and simulate immune cell states. Emerging models are beginning to enable the simulation of genetic perturbations, supporting the prediction of functional effects during disease progression and the prioritisation of potential therapeutic targets.^14^ By linking genetic variation to chromatin accessibility, transcription factor activity, gene expression and downstream protein and metabolic states, population‐scale immune multi‐omics data may further facilitate the transition of virtual cells toward more quantitative and cell‐type‐resolved representations. In this context, PIMAs could provide population‐level context and regulatory depth, although fully bridging genetic associations and immune phenotypes remains an ongoing challenge.

## 3 CHINESE IMMUNE MULTI‐OMICS ATLAS AS AN EXEMPLAR OF INTEGRATED POPULATION IMMUNE PROFILING

The Chinese Immune Multi‐Omics Atlas (CIMA),^10^ recently published in *Science*, exemplifies how PIMAs can extend descriptive immune atlases into integrated, population‐specific reference resources. By profiling over 10 million circulating immune cells from 428 Chinese adults, CIMA combines single‐cell transcriptomic and chromatin accessibility data with WGS and plasma‐level lipidomic, metabolomic, and biochemical measurements.^10^ While population‐scale immune atlas studies such as OneK1K,^7^ AIDA,^9^ FinnGen^15^ and TenK10K^16^, ^17^ have been instrumental in systematically cataloguing transcriptional and regulatory diversity across large cohorts, they reflect diverse study designs that balance cohort size, cellular resolution, and molecular modalities (Figure 1 Top panel). In this context, CIMA places particular emphasis on deep per‐individual cellular profiling (with a median of approximately 15 000 cells for scRNA‐seq and 9600 cells for scATAC‐seq per donor), enabling more comprehensive characterisation of rare immune cell subsets and enhancing statistical power for detecting cell type‐specific regulatory effects. This design facilitates the identification of a broader set of genes and cis‐regulatory elements influenced by genetic variation, thereby strengthening links between genotype, regulatory activity, and immune cell function at the population scale.

**FIGURE 2:**
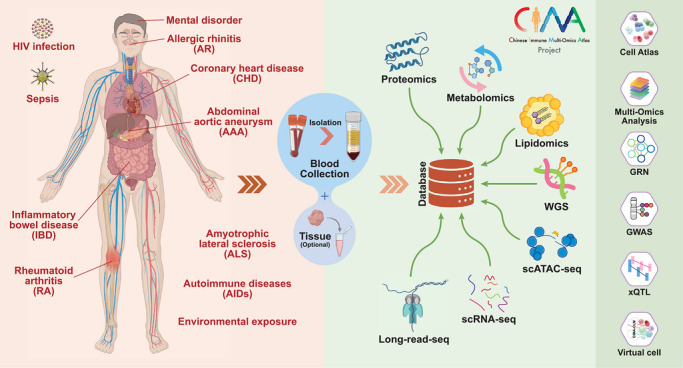
Study design framework of the Chinese Immune Multi‐Omics Atlas (CIMA) Project. (Left panel) Diseases or conditions involved in the CIMA project. (Right panel) Omics technologies and analytical frameworks involved in the CIMA project.

This integrative framework allows CIMA to trace how genetic variation propagates through regulatory networks to influence immune phenotypes (Figure 2). Extensive mapping of 338 036 cis‐regulatory elements and construction of enhancer‐driven gene regulatory networks delineates transcription factor programs that define immune cell identity across fine‐grained cell types, while cell‐type‐resolved quantitative trait locus analyses connect genetic variants to 9600 eGenes and 52 361 caPeaks. These analyses enrich descriptive immune cell maps with regulatory context and population specificity, as illustrated by the asthma‐associated variant rs34415530, which modulates *IKZF4* expression specifically in CD4^+^ FOXP3^+^ regulatory T cells and is linked to IL‐12B secretion differences. Moreover, the development of CIMA‐CLM, a cell language model that predicts chromatin accessibility from DNA sequences and gene expression, demonstrates how population‐scale data can support the construction of predictive virtual cell models capable of evaluating variant effects computationally, transforming static associations into dynamic, mechanistically interpretable frameworks. The CIMA study highlights a major advance in human immunology by showing that population‐scale single‐cell multi‐omics can resolve cell type‐specific regulatory mechanisms underlying genetic effects, thereby enabling more precise links between genetic variation and immune phenotypes.^18^

## 4 TOWARDS A GLOBALLY INTEGRATED AND DISEASE‐EXPANDED PIMA FRAMEWORK

With population‐scale immune atlases now emerging across multiple regions, the central challenge has shifted from establishing feasibility to achieving coordination and interoperability. Efforts such as CIMA, alongside parallel initiatives across Europe, Asia and beyond, collectively demonstrate that population‐specific immune reference frameworks are both technically achievable and biologically informative.^7^, ^9^, ^10^, ^15^, ^16^, ^19^ The next imperative is therefore to organise these independent efforts into a globally integrated framework — one that enables systematic harmonisation of multi‐omics data and immune cell‐resolved regulatory models across populations, rather than isolated interpretation. Central to such a framework is the adoption of shared principles for data generation, processing and multi‐omics integration, allowing virtual cells constructed from different populations to become directly comparable and mutually informative.

Equally important is the extension of this framework beyond healthy population cohorts into disease‐focused settings. The majority of existing PIMAs have been established on predominantly healthy populations, which, while essential for defining regulatory baselines, provides limited direct insight into the mechanisms underlying immune‐mediated disease. Expanding PIMAs to encompass disease cohorts (spanning autoimmune, inflammatory, infectious and oncological conditions) would enable the systematic dissection of how genetic and regulatory variation contribute to immune dysfunction in specific pathological contexts. Such an extension would transform population immune atlases from descriptive reference resources into mechanistically actionable frameworks, ultimately bridging the gap between the regulatory architecture defined in healthy populations and the cellular and molecular perturbations that drive immune‐mediated diseases.
